# M. Cazenave on the Different Sorts of Caustics

**Published:** 1845-07

**Authors:** 


*M. Cazenave on the Different Sorts of Caustics.*—The *Powder of Dupuytren* is composed of one part of arsenious acid and 200 parts of Calomel. It is a mild and very manageable caustic, that is useful in cases of Lupus in women and children, when the ulceration is superficial and of limited extent. If the diseased part be dry, it may be necessary to denude it by means of a blister, and then to sprinkle the powder upon the raw surface. A certain amount of heat and painful swelling is usually caused by this application. When it falls off, there is generally observed a decided modification of the diseased surface. A few applications are sufficient to effect a cure in a great many instances.

The *Vienna powder and paste* are remedies of great power in certain cases of lupous ulceration. They are composed of equal parts of powdered quicklime and potassa cum calce. In using it, we take a portion of this mixture, and add a small quantity of spirits of wine to bring the powder to the consistence of a paste. A piece of adhesive plaster, with a hole in it of the size of the intended eschar, should be laid over the diseased surface, and the paste is then applied upon the exposed part. It is to be left on for ten or twenty minutes, according to the depth of the eschar that is wished, and the ability of the patient to endure the pain.

The *chloruret of zinc paste*, is much used in the present day. It is made by mixing one part of this substance with one or two parts of flour, moistening the mixture with as little water as possible. The pain produced by this application usually lasts for several hours. A greyish-coloured eschar is formed; and this, in most cases, remains attached for two or three weeks before it is separated. The surface underneath is generally not ulcerated. M. Cazenave very frequently has recourse to this caustic in certain cases of lupus, to destroy the non-ulcerated tubercles.

For this purpose, he usually applies only a very thin layer of the paste, so as not to destroy the entire tubercle; and in this manner he often succeeds in effecting a complete resolution of it, without any scar being left behind.

In very many cases of long-standing and deeply-corroding Lupous ulceration, he gives the preference to the Arsenical paste over the two others which we have mentioned: its action is two-fold; local as a caustic; and general, by becoming absorbed, and exercising a potent alterative or modifying influence upon the economy. The following is the formula which he invariably uses:—

Take of White oxyde of arsenic, 2 parts.
Sulphate of mercury, 1 part.
Animal charcoal in powder, 2 parts.

When used, a small quantity of this powder is to be made into a thin paste by the addition of a few drops of water; this is put upon the denuded surface—we should seldom or never exceed in extent that of a franc-piece. This application usually produces not only very sharp pain, but also a severe erysipelatous swelling, which lasts for 24 or 36 hours, and is sometimes accompanied with grave constitutional symptoms. Generally these subside very quickly; and then there remains on the cauterised part a hard brown crust, which often adheres for nearly a month, before it is detached.

